# Establishment and characterisation of a new breast cancer xenograft obtained from a woman carrying a germline *BRCA2* mutation

**DOI:** 10.1038/sj.bjc.6605900

**Published:** 2010-09-28

**Authors:** L de Plater, A Laugé, C Guyader, M-F Poupon, F Assayag, P de Cremoux, A Vincent-Salomon, D Stoppa-Lyonnet, B Sigal-Zafrani, J-J Fontaine, R Brough, C J Lord, A Ashworth, P Cottu, D Decaudin, E Marangoni

**Affiliations:** 1 Preclinical Investigation Unit, Institut Curie – Translational Research Department, Hôpital St Louis, Quadrilatère historique, Porte 13, 1, Ave Claude Vellefaux, Paris 75010, France; 2 Department of Tumor Biology, Institut Curie, Paris, France; 3 INSERM U830, Institut Curie, Paris, France; 4 University Paris Descartes, Paris, France; 5 National Veterinary School of Maisons Alfort, Maisons-Alfort, France; 6 Gene Function Laboratory, The Breakthrough Breast Cancer Research Centre, The Institute of Cancer Research, London SW3 6JB, UK; 7 Department of Medical Oncology, Institut Curie, Paris, France

**Keywords:** BRCA2 mutation, human breast cancer xenograft, preclinical model

## Abstract

**Background:**

The *BRCA2* gene is responsible for a high number of hereditary breast and ovarian cancers, and studies of the BRCA2 biological functions are limited by the lack of models that resemble the patient's tumour features. The aim of this study was to establish and characterise a new human breast carcinoma xenograft obtained from a woman carrying a germline *BRCA2* mutation.

**Methods:**

A transplantable xenograft was obtained by grafting a breast cancer sample into nude mice. The biological and genetic profiles of the xenograft were compared with that of the patient's tumour using histology, immunohistochemistry (IHC), *BRCA2* sequencing, comparative genomic hybridisation (CGH), and qRT–PCR. Tumour response to standard chemotherapies was evaluated.

**Results:**

Histological profile identified the tumour as a basal-like triple-negative breast cancer. Targeted *BRCA2* DNA sequencing of the xenograft showed the presence of the mutation previously identified in the carrier. Comparative genomic hybridisation array profiles of the primary tumour and the xenograft revealed a high number of similar genetic alterations. The therapeutic assessment of the xenograft showed sensitivity to anthracyclin-based chemotherapy and resistance to docetaxel. The xenograft was also highly sensitive to radiotherapy and cisplatin-based treatments.

**Conclusions:**

This study describes a new human breast cancer xenograft obtained from a *BRCA2*-mutated patient. This xenograft provides a new model for the pre-clinical drug development and for the exploration of the drug response biological basis.

Germline *BRCA2* mutations in female carriers confer a cumulative breast cancer risk at age 70 years of 49% (95% CI: 40–57%), an ovarian cancer risk of 18% (95% CI: 13–23%), and a moderate increased risk of pancreatic cancer (Chen and Parmigiani, 2007).

Although the available evidence is not sufficient to decisively conclude that the clinical outcome of women with *BRCA1/2*-associated breast cancer differs significantly from those of women with sporadic tumours, *BRCA1*-associated breast cancer often manifests adverse outcome features.

Establishment of pre-clinical models, which accurately reflect the genetic and phenotypic features of primary tumours, and their response to treatment, is an important step in identifying novel therapeutic targets and testing new treatment modalities. New strategies may take advantage of the specific DNA repair defects inherent in *BRCA*-deficient cells, such as the defect in homologous recombination. In fact, most of the insights into the functions of the BRCA2 protein have included key insights from studies of mice by the use of gene targeting and from studies of altered mouse embryonic cells (Evers and Jonkers, 2006).

BRCA2 has a key role in DNA double-strand break repair and cell-cycle control. BRCA2-related defects are associated with chromosomal abnormalities, a hallmark of the genomic instability that could foster tumourigenesis. Moreover, BRCA2 participates in the regulation of mitosis and cytokinesis that contribute to numerical chromosomal stability.

Although conventional, non-conditional, mouse mutants might be used to model familial forms of cancer, they do not mimic sporadic tumourigenesis because the initiating mutation is present in all cells of the body, including those that constitute the tumour microenvironment.

Moreover, embryonic lethality and development of non-epithelial tumours are another important limitation of genetically mutated *Brca2* mice. Some murine *Brca2* mutant mammary tumour models develop mammary tumours with histopathological features that are significantly different to their human counterparts. Although some studies report a strikingly similar histopathology in *BRCA1* null breast tumours from mice and humans (Dennis, 1999; Xu *et al*, 1999), their relevance to the human situation remains to be demonstrated. The most used human *BRCA2*-mutated model is the CAPAN1 pancreatic cell line that is mainly used to understand drug resistance in *BRCA1/2* mutation carrier, as well as in defining functionally important domains within BRCA2 (Edwards *et al*, 2008). To date, only one *BRCA2*-mutated breast cancer xenografts (MX1) is available, (Donawho *et al*, 2007) but its characterisation has not been described in detail.

Genetic testing to identify *BRCA1/2* mutations is widely available and commonly employed. As a result, increasing numbers of women are aware that they are mutation carriers at the time of their cancer diagnosis. Unfortunately, current knowledge is not sufficient to mandate specific local or systemic treatments that are tailored to *BRCA1/2* mutation carriers. In fact, the available studies examining the issue of whether *BRCA1/2*-associated breast cancer should be treated differently from sporadically occurring, non-familial disease are almost exclusively retrospective and limited by small size and various ascertainment biases.

Recently, inhibitors of the DNA repair proteins, poly(ADP-ribose) polymerase 1 and 2 (PARP1/2), have been shown to be selectively cytotoxic to tumour cells with BRCA1 or BRCA2 deficiency. Preclinical data, including that generated with a limited array of tumour cell line xenografts, suggest that PARP inhibitors can act as single agents to selectively kill cancers with BRCA1 or BRCA2 mutations, and phase I clinical trial results confirm that PARP inhibitors have some single-agent activity in cancers with *BRCA1/2* mutations (Fong *et al*, 2009). However, little is known about the long-term effects of these drugs and it seems likely that some tumours may have *de novo* resistance or acquired resistance (Ashworth, 2008). Thus, definitive answers remain elusive, and preclinical evaluation of new targeted therapy is limited by the lack of suitable preclinical models (Robson, 2007a).

Here, we report the establishment and characterisation of a novel xenograft, human breast cancer xenograft (HBCx-17) established from a breast cancer in a woman carrying a *BRCA2* germline mutation. We show that this xenograft accurately reflects the genetic and the phenotypic features of the primary tumour, thus providing a new model to test new therapies for *BRCA2*-mutated patients.

## Materials and methods

### Animals and establishment of tumour xenografts

The breast cancer specimen was obtained with informed consent from the patient undergoing surgery. Fresh tumour fragments were grafted into the interscapular fat pad of 8–12-week-old female Swiss nude mice, under avertin anaesthesia. Mice were maintained in specific pathogen-free animal housing (Institut Curie, Paris, France) and received oestrogen (8 *μ*g ml^−1^) diluted in drinking water. Xenografts appeared at the graft site about 1 month after grafting. One xenograft was subsequently transplanted from mouse to mouse and stocked frozen in DMSO-fetal calf serum solution or frozen dried in nitrogen for further studies, and a fragment was fixed in phosphate-buffered saline (PBS) 10% formol for histological studies. The experimental protocol and animal housing were in accordance with institutional guidelines as put forth by the French Ethical Committee (Agreement B75-05–18, France).

### Histology and IHC

The morphology of patients’ tumour tissue and of the xenograft was compared using paraffin-embedded sections and standard protocols (Vincent-Salomon *et al*, 2007). Tumours were removed from mice and immediately fixed in a 10% formol/PBS solution.

Determination of oestrogen receptor, progesterone receptor, ERBB2, Ki67, cytokeratin (CK) 5/6, and epidermal growth factor receptor (EGFR) status by IHC was done according to previously published protocols (Vincent-Salomon *et al*, 2007).

To search for spontaneous lung metastasis of the HBCx-17 xenograft, mice were killed when the tumour reached an ethical size (about 2500 mm^3^), and lungs were formalin-fixed *in-toto* for histological evaluation.

### Compounds and therapeutic assays

Doxorubicin, 2 mg kg^−1^ (Adriamycin, Teva Pharmaceuticals, Paris, France), and cyclophosphamide, 100 mg kg^−1^ (Endoxan, Baxter, Maurepas, France), diluted in 0.9% NaCl, and docetaxel, 20 mg kg^−1^ (Taxotere, Sanofi-Aventis, Paris, France), diluted in its specific excipient, were given by intraperitoneal (i.p.) route at 3-week intervals. Ifosfamide, 90 mg kg^−1^ (Holoxan, Baxter) was given by i.p. route on three consecutive days every 3 weeks. Cisplatin, 1 mg kg^−1^ (Mylan, Hoeilaart, Belgium) was diluted in 0.9% NaCl and given weekly by i.p. route. Capecitabine (Xeloda, Roche, Basel, Switzerland), 540 mg per kg per day was diluted in glucose 5% and given per os in two administrations a day. Radiotherapy was administered locally with one dose of 8 Gy or two weekly fraction doses of 8 and 7 Gy, respectively, for a total dose of 15 Gy. Irradiation experiments were done using a caesium source having a dose rate of 2.15 Gy per min.

Therapeutic assessments were performed as described elsewhere (Marangoni *et al*, 2007). Briefly, tumour volume was calculated as *V*=*axb*^2^/2, *a* being the largest diameter and *b* the smallest. Treatment was initiated when tumours in each group achieved an average volume of ∼170–200 mm^3^. For each tumour, *V*s were reported to the initial volume as relative tumour volume (RTV). Means (and s.e.) of RTV in the same treatment group were calculated, and growth curves were established as a function of time. Optimal tumour growth inhibition (TGI) of treated tumours *vs* controls was calculated as the ratio of the mean RTV in treated group to the mean RTV in the control group at the same time. Statistical significance of TGI was calculated by the paired Student's *t*-test, by comparing the individual RTVs in the treated and control groups. Mice were ethically killed when the tumour volume reached about 2500 mm^3^.

### DNA sequencing

Screening for *BRCA2* point and small size mutations was performed through analysis of genomic DNA from patient's tumour. In all, 100 ng of DNA was amplified using Taq ABgene 0.025 U *μ*l^−1^ (ABgene UK, ref: http://www.abgene.com), 4 × 0.2 mM dNTPs, 1.5 mM MgCl_2_, and 0.3 *μ*M of each primer in a final reaction volume of 50 *μ*l. Amplification was performed with an initial denaturation step at 94°C for 5 min followed by 35 PCR cycles: denaturation at 94°C for 30 s, annealing at 54°C for 30 s, and elongation at 72°C for 30 s.

PCR products were separated by agarose gel electrophoresis, purified (Macherey Nagel, Düren, Germany), and sequenced using one of the PCR primers (usually the forward primer, except in the case of poor sequence quality). Big Dye Cycle Sequencing Reactions and an ABI3130XL automated sequencer (Applied Biosystems, Foster City, CA, USA). Seqscape (Applied Biosystems) software was used for sequence analysis.

### Genotypage

Allelic loss was analysed by amplification of two microsatellite markers flanking the *BRCA2* gene: D13S260 and D13S1701. Germline DNA, obtained from a blood sample, and tumour and xenograft DNA were compared. In all, 100 ng of DNA was amplified using AmpliTaq Gold 5 U *μ*l^−1^ (Applied Biosystems), 4 × 0.2 mM dNTPs, 1.5 mM MgCl_2_, and 0.3 *μ*M of each primer in a final reaction volume of 10 *μ*l. Amplification was performed with an initial denaturation step at 94°C for 5 min followed by 30 PCR cycles: denaturation at 94°C for 30 s, annealing at 55°C for 30 s, and elongation at 72°C for 30 s. A total of 1 *μ*l of PCR products was mixed with 19 *μ*l of Formamide Hi-Di (Applied Biosystems) and 0.5 *μ*l of Genescan 400 ROX Size Standard (PE Applied Biosystems) and separated on ABI3130XL automated sequencer (Applied Biosystems). Genemapper (Applied Biosystems) software was used for genotype analysis.

### Quantitative multiplex PCR of short fluorescent fragments

Quantitative multiplex PCR of short fluorescent fragments is a sensitive method for the detection of large gene deletions or duplications. It is based on the simultaneous amplification of short genomic fragments using dye-labelled primers under quantitative conditions (Casilli *et al*, 2002; Tournier *et al*, 2004). PCR products were analysed on a sequencing platform used in the fragment analysis mode, where both peak heights and areas are proportional to the quantity of template present for each target sequence. Nine amplicons of *BRCA2* between 180 and 300 bp were amplified in the same multiplex reaction. As an internal control, we included in each reaction a fragment of different gene in which deletion was not expected (MLH1 exon 14). One primer from each primer pair was 5′-labeled with 6-FAM fluorochrome.

In all, 100 ng of two genomic DNA from the xenograft, two normal control, and one *BRCA2*-mutated control were amplified in a final volume of 25 *μ*l, including 0.06–0.24 *μ*mol l^−1^ for each primer and 12.5 *μ*l of QIAGEN master mix (QIAGEN multiplex PCR Kit). After an initial denaturation step, samples underwent 23 cycles (30 s at 95°C, 40 s at 54°C, and 60 s at 72°C). After the multiplex reactions, the DNA fragments were separated on an ABI3130XL automated sequencer and analysed using Genemapper Software (Applied Biosystems).

For this analysis, we used visual sample-to-control comparison. We estimated allele dosage by superimposing the electropherogram of the tested sample onto the corresponding image for a control DNA sample, after adjusting the vertical scale of the internal control amplicon. Allelic losses of one or more amplicons are represented by a two-fold reduction in the intensity (peak heights) of an amplicon of the sample analysed. Allelic duplication of one or more amplicons is represented by a 0.5-fold rise in the intensity (peak heights) of an amplicon of the sample analysed.

### Protein analysis

Whole-cell protein extracts were prepared from tumour samples by homogenising the tissue in RIPA buffer (10 mM Tris-Cl, pH 7.5, 150 mM NaCl, 1 mM EDTA, 1% Nonidet P40, 0.5% sodium deoxycholate, 0.1% SDS, protease inhibitors). Protein concentrations were measured with Bio-Rad Protein Assay Reagent (Bio-Rad, Marnes-la-Coquette, France). Immunoprecipitations were performed by incubating Protein G beads (Sigma, Steinheim, Germany), 1–2 mg of precleared cell lysate, and anti-BRCA2 mouse monoclonal Ab-1 antibody (dilution 1 : 200; Merck) overnight at 4°C. Beads were subsequently washed three times in cold lysis buffer, after which 2 × loading buffer was added and the samples were boiled for 2 min before SDS–PAGE. For western blotting analysis, lysates were subjected to electrophoresis on Novex precast gels (Invitrogen, Cergy Pontoise, France) and immunoblotted overnight at 4°C with the following antibodies: anti-BRCA2 and anti-*β*-tubulin, T4026 (Sigma). This was followed by incubation with anti-IgG-horseradish peroxidase and enhanced chemiluminescent detection (GE Healthcare Bio-Sciences AB, Uppsala, Sweden). As a control, full-length BRCA2 was detected in lysates generated from 293T cells.

### Detection of ER*α*, Ki67, and ERBB2 by quantitative RT–PCR

Total RNA extraction and cDNA synthesis were done as previously described from 1 *μ*g total RNA (de Cremoux *et al*, 2004). ER*α*, Ki67, and ERBB2 transcripts were quantified using real-time quantitative reverse transcription–PCR (RT–PCR) assays. The nucleotide and probe sequences, and the conditions of PCR have been previously described (de Cremoux *et al*, 2007). Results were expressed as *N*-fold differences in target gene expression relative to a reference gene defined as ‘N target’.

### Array-based CGH

A genome-wide resource of 5244 fluorescence *in situ* hybridisation mapped, sequenced BAC and PAC clones, verified for gene and marker content were represented as immobilised DNA targets on glass slides for array-based CGH analysis, allowing a mean resolution of 0.5 Mb all along the genome. Each clone was spotted in quadruplicate on a slide prepared by Integragen (Evry, France) and developed by the Institut National de la Sante et de la Recherche Medicale Unit U830. After extraction, 1.5 *μ*g of each test and control DNA samples were digested with *Dpn*II enzyme (Ozyme, Saint-Quentin en Yveline, France) and purified with a QIAquick PCR purification kit (Qiagen, Courtaboeuf, France). They were then labelled by random priming using a Bioprime DNA labelling kit (Invitrogen) with the appropriate cyanine dye (Cy3 or Cy5; PerkinElmer, Waltham, MA, USA). The control and test DNA were coprecipitated with Cot-1 DNA (Invitrogen), denatured, and resuspended in hybridisation buffer (50% formamide). Competitive cohybridisation was done on CGH array slides. After 24-h hybridisation, slides were washed with SDS and saline citrate, dried, and scanned using a 4000B scan (Axon, Orleans, CA, USA). Image analysis was done with Genepix 5.1 software (Axon) and processed using a software developed at the Curie Institute (La Rosa *et al*, 2006). Any BAC with less than two replicates flagged for not fulfilling qualitative spot criteria was excluded. A ratio <0.8 was considered as a loss, a ratio >1.2 was considered as a gain, and a ratio >1.5 was considered as amplification (Auger *et al*, 2006).

Data analysis was based on the normalised ratios of Cy5/Cy3 signals observed for each BAC clone that previously passed the flag assessment procedure. For autosomal chromosomes, the loss of a given locus was defined by a ratio ⩽0.8, a gain was defined by a ratio ⩾1.2 and <2.0, and an amplicon was defined by a ratio ⩾2.0. For X chromosomes, a loss was defined by a ratio ⩽1.2, a gain was defined by a ratio ⩾1.7, and an amplicon was defined by a ratio ⩾2.5. The data analysis was done according to previously published protocols (Vincent-Salomon *et al*, 2007).

### Clinical features of the patient

The *BRCA2* mutation carrier from whom the HBCx-17 xenograft was obtained was a 37-year-old woman who was affected at 32 years of age for a first invasive ductal carcinoma (IDC) of grade III that was treated with surgery, FEC100, and radiotherapy. After 6 years, she developed a contralateral IDC and was treated by upfront surgery followed by six cycles of docetaxel and radiotherapy. The xenograft was established from the second carcinoma. The patient tumour was C-ERBB2, oestrogen-, and progesterone-receptor negative, with a high mitotic index. The patient had a strong familial history of breast and ovarian cancer. *BRCA1/2* gene testing, performed with the informed consent of the patient, identified a mutation in the *BRCA2* gene: c.6033_6034delTT; p.ser2012GlnfsX5.

## Results

### Histology of xenograft and comparison with patient tumour

**Figure 1:**
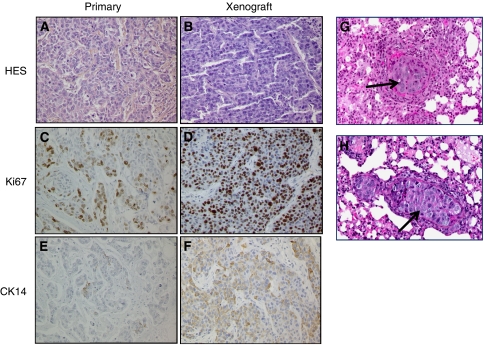
Representative haematoxylin-and-eosin-stained sections of patients and xenografts tumours. Haematoxylin and eosin sections Gx100 (**A** and **B**), KI67 (**C** and **D**), and CK14 (**E** and **F**). Lung metastases are shown in pictures **G** and **H** (arrows) clusters of tumour cells obstruct the lumen of a small number of pulmonary arterioles (cancerous emboli), without evident effraction of the arteriolar media.

Histopathological analysis was performed with the primary tumour and the xenograft HBCx-17 at passage 6. As shown in Figure 1A and B, the histology of the original tumour was conserved in the xenograft. Indeed, HBCx-17 showed an infiltrating ductal carcinoma with typical loss of tubule formation, prominent nuclear pleomorphism, and mitotic activity. Irregular infiltration of stroma was observed in both patient and xenografts. Assessment of tumour proliferation using Ki67 staining showed a high proliferative rate in the primary tumour, which was increased in the xenograft (Figure 1C and D). Both primary and xenograft tumours were negative for CK5 and CK6 (data not shown) but positive for CK14 expression as shown in Figure 1E and F. Both tumour sample and HBCx-17 are negative for ERBB2 and oestrogen, and progesteron receptor, and the clinical sample presented a strong EGFR staining that the xenograft did not (data not shown).

To search for spontaneous lung metastasis of the HBCx-17 xenograft, mice lungs were analysed by histochemistry. Figure 1G and H show two examples of small metastasis: clusters of tumour cells obstructed the lumen of a small number of pulmonary arterioles (cancerous emboli), without evident effraction of the arteriolar media. Lung metastases were detected in 23% of mice (six positive animals on 26).

### CGH array and genomic alterations

**Figure 2:**
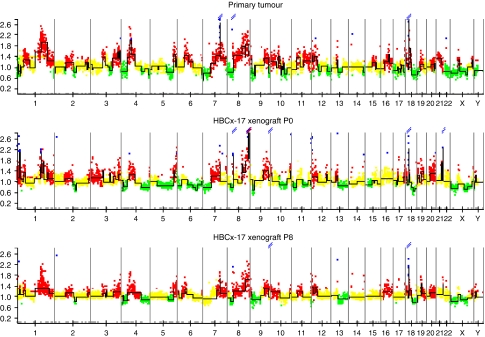
HBCx-17 tumour array CGH profiling of patient (top) and xenograft (bottom). Loss (green points), gain (red points), or amplification (blue points) of chromosome material. Recurrence of copy number alterations (*y* axis) is plotted for each probe aligned along the *x* axis in chromosome order.

The clinical sample and xenografted tumours at passages 0, 6, and 8 were characterised for genetic parameters using CGH array technique (Figure 2). Comparative genomic hybridisation array analysis showed a very high number of alterations and quite similar gene copy number changes. The genomic profiles of the primary tumour and different passages in the xenograft were very similar, sharing large regions of DNA amplifications. Common DNA gains and deletions are described in the Tables 1 and 2. These amplifications are described in the Table 1 such as amplicons of chromosome 1q and 8p.

### Gene expression profiles

The histological classification of the HBCx-17 as a triple-negative tumour was confirmed by quantitative RT–PCR analysis of the ER, PR, and ERBB2 receptors (Table 3). Both the clinical sample and the xenograft tumours were negative for the three receptors. By contrast, the basal-like CK5 and CK6 were not expressed, but the classification of basal-like breast cancer was done on CK14 expression as determined by IHC (Figure 1).

The *TP53* status was found to be mutated in the xenograft tumour as defined by the functional Fasay Assay (data not shown).

### *BRCA2* alterations

**Figure 3:**
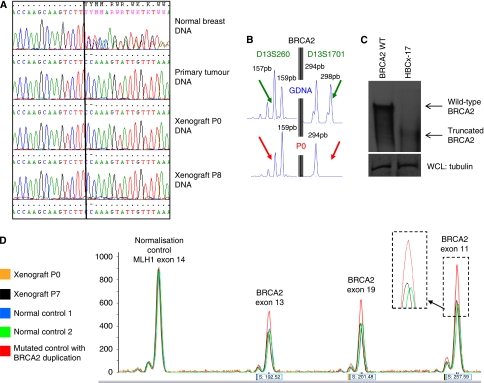
(**A**) Chromatograms of tumour and normal breast tissue DNA showing *BRCA2* mutation: (c.6033_6034delTT/p.Ser2012GlnfsX5) Germline DNA (GDNA) shows both mutated and wild-type alleles. Loss of the wild-type allele with retention of the mutant one is shown in the primary tumour and in passage 0 (P0) and 8 (P8) of the xenograft. (**B**): Amplification of two microsatellites (D13S260 and D13S1701) flanking *BRCA2* shows loss of heterozygosity in P0. (**C**) Illustrates an anti-BRCA2 immunoprecipitation on lysates generated from tumour sample HBCX-17 and 293T cells. Western blot analysis detected full-length BRCA2 in the 293T samples and a truncated BRCA2 product of the predicted size in the tumour sample. (**D**) quantitative multiplex PCR of short fragment shows that mutated allele is duplicated whereas wild-type allele is lost.

DNA sequencing of the primary tumour, and P0 and P8 from the HBCx-17 xenograft showed the presence of the germline *BRCA2* mutation identified in the patient (c.6033_6034delTT; p.Ser2012GlnfsX5; Figure 3A). The informativity of the two studied markers located at the *BRCA2* locus, D13S260 and D13S1701, has allowed detecting the presence of the two alleles at the germline DNA level, whereas the primary tumour and the xenograft DNA showed loss of one allele. Loss of heterozygosity (LOH) at the *BRCA2* locus was confirmed in the xenograft at P0 by using two flanking microsatellite markers for BRCA2, D13S260, and D13S1701 (Figure 3B).

To assess the LOH consequence on the BRCA2 protein, a protein analysis was performed by western blot on cell lysate obtained from a control breast tumour xenograft (having no BRCA2 mutation) and from the HBCx-17 xenograft. As shown in Figure 3C, the BRCA2-mutated xenograft lysate contains a truncated form of the BRCA2 protein and has lost the wild-type protein.

Quantitative multiplex PCR of short fluorescent fragments was used to determine *BRCA2* copy number. Figure 3D shows the electrophoregrams of germline DNA, and P0 and P7 DNA (orange and black). Mutated (*BRCA2* duplicated) DNAs were used as controls (green and red, respectively). Xenograft samples and control samples were perfectly superimposed after normalisation, indicating a duplication of *BRCA2*-mutated allele.

### Tumour responses of xenograft to conventional chemotherapies and radiotherapy

**Figure 4:**
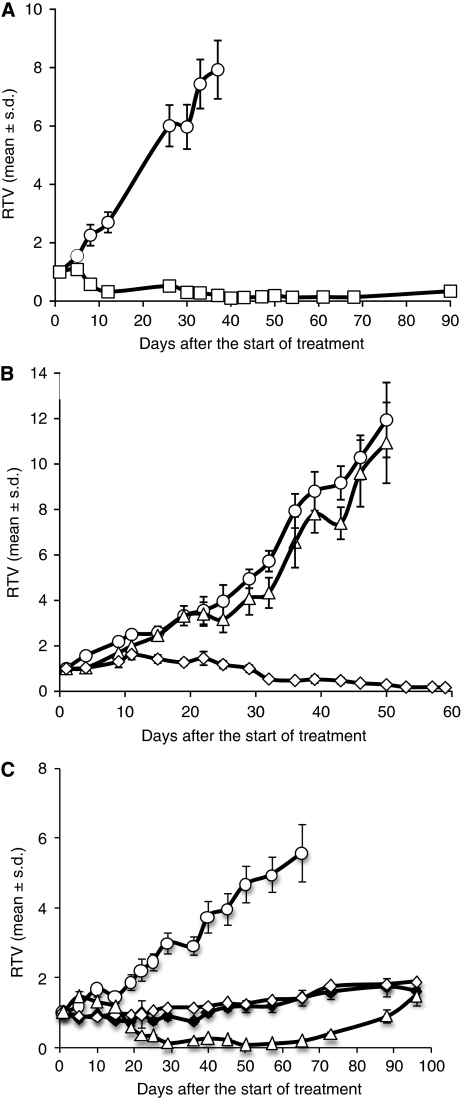
Tumour growth curves of HBCx-17 xenograft as a function of time: HBCx-17 bearing mice were treated with two cycles of AC (**A**), a combination of doxorubicin (2 mg kg^−1^ i.p. every three weeks) and cyclophosphamide (100 mg kg^−1^ i.p. every three weeks), with docetaxel (▵) (20 mg kg^−1^ i.p. every three weeks) or capecitabin (◊) (540 mg kg^−1^ per os 5 days per week two times) (**B**) or with a combination of cisplatin (1 mg kg^−1^ i.p. once a week) and ifosfamide (90 mg kg^−1^, 3 consecutive days every 3 weeks) (▵) compared with 15 Gy (⧫) or 7 Gy (◊) irradiations (**C**). Controls (○) were not treated. Mice were treated at day 1, and tumour volume was measured twice a week. Tumour growth was evaluated by plotting the mean of the RTV±s.d. per group (each group consisted of 10 mice) over time after first treatment.

The HBCx-17 xenograft growth parameters (tumour latency and tumour take) are published (Marangoni *et al*, 2007). Tumour responses to standard chemotherapies used in the treatment of breast cancer and radiotherapy have been studied. As shown in Figure 4A, the HBCx-17 xenograft was a high responder to AC, with five out of eight complete regressions and a TGI of 98%. The HBCx-17 model was also sensitive to capecitabin-based treatment with a TGI of 98% 4 weeks after start of the treatment. No response was observed to docetaxel, as shown in Figure 4B. Cisplatin/ifosfamide combination gave also an important growth inhibition (98%) with 7 out of 10 complete regressions at day 50 (Figure 4C). Radiotherapy assays were done with a caesium irradiation source. The mice received either 8 or 15 Gy locally delivered in two fractions of 7 and 8 Gy. The xenograft was a high responder to irradiation with a TGI of 73 and 75% at 8 and 15 Gy, respectively (Figure 4C).

## Discussion

In this work, we report the characterisation of a human breast tumour xenograft obtained from a woman carrying a *BRCA2* mutation. The basal-like morphology of the patient tumour was conserved in the xenograft including the stroma component and tissular architecture. Ki67 staining was higher in the xenograft's tumour than in the primary tumour, suggesting that tumour engrafting may have selected highly proliferating cancer cells. Studies on large series of *BRCA2*-associated breast cancers indicate that these tumours are predominantly high-grade IDCs of no special marker subtype, and that they are more often oestrogen- and progesterone-receptor positive (Lakhani *et al*, 2002; Brekelmans *et al*, 2007; Palacios *et al*, 2008). As the majority of *BRCA2*-deficient cells, the tumour cells contain only the truncated *BRCA2* protein, indicating that LOH has occurred as a consequence of an inactivating mutation in the second allele. This is what normally happens in most BRCA2-mutated tumour, where inactivation of the wild-type allele occurs by LOH, abolishing normal protein expression (Smith *et al*, 1992; Collins *et al*, 1995). In addition, multiplex PCR of short fragment showed that loss of the wild-type gene was associated with duplication of the mutated *BRCA2* gene. LOH and copy number abnormalities are often associated with *BRCA1*, and *BRCA2*-associated breast or ovarian cancer (Staff *et al*, 2000).

Concordance between the clinical sample and xenograft was also shown by CGH array analysis. Genetic profiles were very similar not only between the patient tumour and the xenografts, but also when comparing xenografts at different passages, suggesting that although extremely altered, the HBCx-17 xenograft genetic profile was stable during subsequent passages. The clinical sample and the xenograft tumours present some of the chromosome alterations that have already been frequently described in the genomic profiles of *BRCA2*-mutated tumours, as gains of 8q and 20q, and loss of 13q (Palacios *et al*, 2008). The amplification of the *MYST3* and *AP3M2* genes was already described like recurrent amplicons associated with reduced survival duration in breast cancer (Chin *et al*, 2006).

Gains in the regions 1q32–q41, 8q22.1–24.3, and 20q12–q13, and loss in the region 8p23.3–p21.2 occur in both the primary and xenograft tumours and have also been found in primary *BRCA2* tumours by different authors (Gronwald *et al*, 2005; Jonsson *et al*, 2005).

However, none of these regions have been confirmed in large number of patients and in studies of independent collections of families, and more extensive studies are necessary to find alterations specific to *BRCA1/2*-mutated tumours.

There is a significant body of preclinical data that supports the hypothesis BRCA1/2-deficient cells are more sensitive to certain chemotherapy agents than are the cells with intact BRCA1 and BRCA2 proteins (Foulkes, 2006). In line with this, the HBCx-17 xenograft showed a pronounced sensitivity to anthracycline-based chemotherapy with >50% of mice showing complete response (no tumour recurrence), as well as to cisplatin and ifosfamide combination. Although *BRCA2* deficiency has not been extensively studied, some previous works showed that decreased BRCA2 function is associated with increased *in vitro* sensitivity to cisplatin, mitomycin, doxorubicin, and etoposide (Foulkes, 2006; Robson, 2007a). The HBCx-17 xenograft was highly resistant to docetaxel. Although some experiments raise the possibility that *BRCA1*-deficient cells may be resistant to anti-cancer agents targeting the microtubules (such as vinca alkaloids and taxanes), no preclinical *in vivo* studies have ever demonstrated taxane resistance in BRCA2-deficient cells (Foulkes, 2006; Robson, 2007b). In the same way, some recent clinical analyses suggest that primary resistance to docetaxel-based chemotherapy correlates with *BRCA1* mutations’ high frequency. No indications exist for breast tumours occurring in *BRCA2* mutation carrier. Our data suggest that, like *BRCA1*, wild-type *BRCA2* could be required for *in vivo* response to mitotic spindle poisons and that the docetaxel resistance could be attributed to *BRCA1/2* involvement in the taxane-induced stress response pathway.

In the clinic, the available evidence is not sufficient to conclude that BRCA1/2-associated breast cancer is differentially sensitive to specific conventional chemotherapeutic agents. In the adjuvant setting, the choice of treatment regimen is not modified based on the presence of such a predisposition. Clinical trials are in progress to further address this issue. Nevertheless, the outcome of hereditary breast cancer remains poor, addressing the questions of potential new strategies that take advantage of the specific DNA repair defects inherent in BRCA-deficient cells. The inhibition of PARP1 potentiates the activity of DNA-damaging agents, such as alkylating drugs, platinums, topoisomerase inhibitors, and radiation in *in vitro* and *in vivo* models. Tumours with DNA repair defects, such as those arising from patients with BRCA1/2 mutations, are more sensitive to PARP inhibition (Bryant *et al*, 2005; Farmer *et al*, 2005; Fong *et al*, 2009). In this context, the HBCx-17 model could improve preclinical assays of PARP inhibitors that are usually done in *BRCA1/2* knockout mice or in pancreatic cancer cells. Different agents are undergoing phase I and II clinical trials in BRCA1/2-associated breast and ovarian cancer, and new compounds are entering in the early preclinical settings. In this perspective, the establishment of this new *BRCA2* breast cancer xenograft reproducing, over successive generations, the patient's characteristics in terms of histology, genetic profile, and biological characteristics may contribute to the preclinical development of innovative therapeutics regimens.

## Figures and Tables

**Table 1:** Recurrent amplicons observed in the primary tumour and maintained in at least two passages of the xenograft

**Cytogenetic region**	**Start position** *	End position*	**Candidate genes**	**Maximum xenograft ratio (passage)**
1q23.3–24.1	163.3	164.3	*LMX1A, RXRG, LRRC52, MGST3, ALDH9A1, TMCO1, UCK2*	2.1 (p8)
7q31.1	111.0	112.4	*DOCK4, IFRD1, NPM1P14, TMEM168*	2.2 (p0)
8p11.21	41.6	42.2	*MIRN486, ANK1, MYST3, AP3M2, PLAT, IKBKB*	2.12 (p8)
13q14.11–12	43.0	44.6	*CCDC122, TSC22D1, NUFIP1*	2.69 (p0)
18q11.2–12.1	19.6	24.3	*CABYR, OSBPL1A, HRH4, RAC1P1, hCG1643695, SS18, PSMA8, TAF4B, KCTD1, hCG38400, AQP4, CHST9, CDH2*	3.3 (p0)

* UCSC Genome Browser on Human May Assembly; positions are given in megabased.

**Table 2:** Recurrent losses observed in the primary tumour and maintained through passages of the xenograft

**Cytogenetic region**	Start position*	End position*	**Candidate genes**
1p34.3	34.4	34.9	*MIRN552*
2q22.2	141.5	142.1	Not known
4p16.1–15.33	8.5	12.3	*GPR78, CPZ, DUB4, DEFB131, DRD5, SLC2A9, MIST, MIRN572, HS3ST1*
4p15.2	25.1	26.5	*SLC34A2, RBPJ, CCKAR, TBC1D19, STIM2*
4q35.1	186.8	189.0	*SORB2, TLR3, CYP4V2, KLKB1, F11, MTNR1A, MRPS36P2*
7p22.2–7p22.1	3.1	5.9	*SDK1, FOXK1, RADIL, PAPOLB, MMD2, RBAK, WIPI2, SLC29A4, FBXL18, MIRN589, ACTB, EGID-654231*
7p15.3	20.2	22.6	*ITGB8, ABCB5, SP8, SP4, DNAH11, CDCA7L, RAPGEF5, MGC87042*
7p15.1	28.3	29.9	*CREB5, CPVL, CHN2, PRR15, WIPF3*
	30.1	31.1	*MIRN550-1, NOD1, GARS, CRHR2, INMT, AQP1, GHRHR, ADCYAP1R1*
7p14.3–14.1	32.5	43.0	*MIRN550-2, KBTBD2, FKBP9, NT5C3, RP9, BBS9, BMPER, AAA1, NPSR1, TBX20, MERPUD2, hCG1642425, SEPT7, ANLN, AOAH, ELM01, GPR141, TXNDC3, SFRP4, STARD3NL, T cell receptor gamma variable genes, AMPH, RALA, CDC2L5, INHBA, GLI3, TCP1L1, PSMA2, MRPL32*
7p12.3	46.0	47.1	*EPS15L2*
8p21.2	22.9	24.0	*TNFRSF10C, TNFRSF10D, TNFRSF10A, CHMP7, R3HCC1, LOXL2, ENTPD4, SLC25A37, NKX3.1, NKX2.6, STC1*
	25.2	25.5	*GNRH1, KCT9, CDCA2*
	25.7	26.3	*EBF2, PPP2R2A, SDAD1P1*
8p21.1	27.4	28.6	*GULOP, CLU, SCARA3, CCDC25, PBK, PNOC, FBXO16, EXTL3*
	28.8	30.0	*KIF13B, DUSP4, MAP2K1P1*
	31.9	32.1	*NRG1*
9p21.3	20.3	24.7	*MIRN491, PTPLAD2, IFNB1, IFNW1, IFNxxx…, LEIF-M, MIRN31, MTAP, CDKN2A, CDKN2B, DMRTA1, ELAVL2*
9p21.2–21.1	27.3	28.9	*IFNK, LING02*
10q22.1	72.1	74.2	*SGPL1, PCBD1, SLC29A3, CDH23, PSAP, CHST3, SPOCK2, ASCC1, SSIT4, DNAJB12, CBARA1*
10q23.31	89.7	91.4	*LIPJ, LIPF, LIPK, LIPN, LIPM, ANKRD22, STAMBPL1, ACTA2, FAS, CH25H, IFIT2, LIPA*
10q23.33–24.1	96.5	98.6	*CYP2C19, CYP2C9, CYP2C8, PDLIM1, SORBS1, ALDH18A1, TCTN3, ENTPD1, CC2D2B, CCNJ, BLNK, DNTT, TMEM10, TLL2, TM9SF3, PIK3AP1, MIRN606*
11q25	131.7	133.7	*OPCML, SPATA19, IGSF9B, JAM, PTP4AP2, NCAPD3, THYN1, ACAD8, GLB1L3*
17p11.2	19.9	21.6	*SPECC1, COTL1P2, MEIS3P2, RNASEH1P1, HSP22, DHRS7B, TMEM11, MAP2K3, KCNJ12*
18q21.2–21.33	46.9	61.0	*DCC, MBD2, POLI, STARD6, C18orf54, C18orf26, RAB27B, CCDC68, TCF4, AC009271.7, TXNL1, WDR7, AC100775.3, ST8SIA3, ONECUT2, FECH, NARS, ATP8B1, AC022724.8, AC090324.7, NEDD4L, ALPK2, AC104971.5, MALT1, AC104971.5, ZNF532, SEC11L3, GRP, RAX, CPLX4, LMAN1, CCBE1, PMAIP1, AC090377.15, MC4R, AC010928.7, CDH20, RNF152, PIGN, KIAA1468, TNFRSF11A, ZCCHC2, PHLPP, AC015989.11, BCL2, FVT1, VPS4B, SERPINB5, AC036176.8, SERPINB12, SERPINB13, SERPINB4, SERPINB3, SERPINB11, SERPINB7, SERPINB2, SERPINB10, SERPINB8*
18q22.3	66.9	68.6	*CBLN2*
	68.8	72.8	*FBXO15, CYB5A, FAUP1, CNDP2, CNDP1*

* UCSC Genome Browser on Human May Assembly; positions are given in megabased.

**Table 3:** Gene expression analysis of ERBB2, KI67, and ER*α* evaluated by quantitative RT–PCR (overexpression limit of 1000 units)

	**ERBB2**	**Ki67**	**ER*α***
Patient tumour	8166 (−)	6788 (++)	34 (−)
Xenograft tumour	10 533 (−)	16 695 (+++)	6 (−)
